# Statistical Approaches for Assessing Health Effects of Environmental Chemical Mixtures in Epidemiology: Lessons from an Innovative Workshop

**DOI:** 10.1289/EHP547

**Published:** 2016-12-01

**Authors:** Kyla W. Taylor, Bonnie R. Joubert, Joe M. Braun, Caroline Dilworth, Chris Gennings, Russ Hauser, Jerry J. Heindel, Cynthia V. Rider, Thomas F. Webster, Danielle J. Carlin

**Affiliations:** 1 National Institute of Environmental Health Sciences, National Institutes of Health, Department of Health and Human Services, Research Triangle Park, North Carolina, USA; 2 Department of Epidemiology, Brown University, Providence, Rhode Island, USA; 3 Department of Preventive Medicine, Icahn School of Medicine at Mount Sinai, New York, New York, USA; 4 Departments of Environmental Health and Epidemiology, Harvard T.H. Chan School of Public Health, Boston, Massachusetts, USA; 5 Department of Environmental Health, Boston University School of Public Health, Boston, Massachusetts, USA

## Abstract

Quantifying the impact of exposure to environmental chemical mixtures is important for identifying risk factors for diseases and developing more targeted public health interventions. The National Institute of Environmental Health Sciences (NIEHS) held a workshop in July 2015 to address the need to develop novel statistical approaches for multi-pollutant epidemiology studies. The primary objective of the workshop was to identify and compare different statistical approaches and methods for analyzing complex chemical mixtures data in both simulated and real-world data sets. At the workshop, participants compared approaches and results and speculated as to why they may have differed. Several themes emerged: a) no one statistical approach appeared to outperform the others, b) many methods included some form of variable reduction or summation of the data before statistical analysis, c) the statistical approach should be selected based upon a specific hypothesis or scientific question, and d) related mixtures data should be shared among researchers to more comprehensively and accurately address methodological questions and statistical approaches. Future efforts should continue to design and optimize statistical approaches to address questions about chemical mixtures in epidemiological studies.

## Background

There is great interest in quantifying the impact of exposure to environmental chemical mixtures on human health. As shown in biomonitoring studies, children and adults are exposed to a large number of environmental chemicals across the life span (Aylward et al. 2013; CDC 2012; Exley et al. 2015; Frederiksen et al. 2014). Many are potentially toxic, but little is known about health effects from exposure to complex mixtures (Carlin et al. 2013; Claus Henn et al. 2014; Goodson et al. 2015; Grandjean and Landrigan 2014; Johns et al. 2012). By examining chemical mixtures, instead of one chemical at a time, it may be possible to more accurately identify risk factors for diseases with environmental origins and develop more targeted public health interventions.

In 2011, the National Institute of Environmental Health Sciences (NIEHS) hosted a workshop on chemical mixtures entitled “Advancing Research on Mixtures: New Perspectives and Approaches for Predicting Adverse Human Health Effects.” This workshop brought together experts from epidemiology, toxicology, exposure science, risk assessment, and statistics to identify key challenges in mixtures research and to suggest approaches for addressing those challenges (Carlin et al. 2013). An important theme that emerged was the need for further collaboration between experts that would help bridge the gap between toxicological and epidemiological studies that involve chemical mixtures. This cross-disciplinary collaboration is a necessary step in understanding exposure to real-world mixtures and the associated health effects. Another key concept that came from the workshop included the need to develop novel statistical approaches that would predict and evaluate effects associated with exposure to mixtures. In addition, the NIEHS has incorporated into its 2012–2017 Strategic Plan (Goal 4) the need for further study of the health effects associated with combined exposures (NIEHS 2012; see http://www.niehs.nih.gov/about/strategicplan/). This goal includes the assessment of joint action of multiple environmental exposures, including chemicals, nonchemical stressors (e.g., socioeconomic, behavioral factors), infectious agents, the microbiome, and nutritional components on toxicity and disease. Moreover, there is a need to identify interactions resulting from combined exposures, determine how the combined exposures affect human health outcomes, and identify preventive measures to mitigate the potential impact of these exposures.

## Objectives

To follow up on the themes from the 2011 workshop, and in an effort to focus on statistical approaches for multi-pollutant (i.e., mixtures) epidemiology studies, the NIEHS convened another workshop in July 2015. This workshop—“Statistical Approaches for Assessing Health Effects of Environmental Chemical Mixtures in Epidemiology Studies”—was designed to bring together experts from the fields of environmental epidemiology and biostatistics (NIEHS 2015). The primary objective of the workshop was to identify and compare different approaches and methods for analyzing chemical mixture data in epidemiological studies.

An innovative approach was used to attract and engage potential workshop participants and conduct a working meeting. This approach involved having participants apply various statistical methods to two simulated data sets and one real-world data set before the workshop. Each data set included a single continuous health outcome (*Y*), multiple chemical exposures, and additional non-exposure variables (e.g., potential confounders). Experts were offered an opportunity to test their statistical methods of choice on the data sets and later exhibit their findings at the workshop.

The first step in the process was to make the simulated data sets available to potential participants approximately six months before the workshop. These data sets have since been made publically available on the NIEHS web site (http://www.niehs.nih.gov/about/events/pastmtg/2015/statistical/). Participants were asked to analyze the data sets using their specific statistical approach(es) and to submit an abstract describing their approach(es). Second, the real-world data set was made available to those who submitted abstracts based on their analyses of the simulated data sets. The methods used to create the two simulated data sets were known only by the workshop organizers and were revealed to those participants that had submitted their analyses to the workshop organizers prior to the workshop. This allowed potential participants to compare their results to the known (i.e., “truth”) results and to reflect on why their results may have differed.

The planning committee received 33 abstracts from academia, government, and industry. Based on these abstracts, subsets of individuals were invited to present their approach and statistical model(s) at the meeting.

## The Data Sets

Simulated data set 1 (*n* = 500) was designed to represent a prospective cohort epidemiologic study with seven continuous, log-normal exposures and one binary variable stipulated to be a confounder that required adjustment. Assumptions were built into the data set and included no loss to follow up, missing or censored data, mismeasurement of the variables, or other potential biases. It was also assumed that the seven exposure variables and the binary variables were neither intermediate variables nor colliders. The data sets were designed such that there were high correlations between exposures, the binary variable was a strong confounder, and directions of effect for the exposures differed. Random, normally distributed noise was added to the outcome variable, and only part of the variation in the outcome was explained by the independent variables. In addition, this data set had fewer exposure variables than the second simulated data set and smaller amounts of unexplained variation (e.g., random noise), non-linear exposure–response functions, and interactions between exposures.

Simulated data set 2 (*n* = 500) represented data from a cross-sectional study of 14 exposure variables. This data set included three potential confounders (two continuous and one binary), a strong correlation between exposures, and strong effect measure modification by a binary confounder (e.g., sex). The exposure variables had complex correlations based on real-world biomarker data from the National Health and Nutrition Examination Survey (NHANES). The second simulated data set featured more exposure variables and more unexplained variation than the first simulated data set, but contained linear exposure–response functions and no interactions between exposures. To understand the nature of the challenges presented to the workshop participants, the reader can find additional information regarding the complexity of the simulated data sets and the assumptions that were built into the data sets on the workshop web site (http://www.niehs.nih.gov/about/visiting/events/pastmtg/2015/statistical/).

The third data set was a modified real-world data set (*n* = 270) that came from a prospective pregnancy and birth cohort study of mothers and children where the results (i.e., truth) were unknown (Braun et al. 2016b). This data set included 22 exposure variables: 14 polychlorinated biphenyls (PCBs), 4 polybrominated diphenyl ethers (PBDEs), and 4 organochlorine pesticides. The outcome consisted of scores on the Mental Development Index (MDI; a measurement of cognition) (Bayley 1969) at ages 1–3 years; covariates included child’s sex and mother’s age, education, race, and smoking status during pregnancy.

For each analysis, workshop participants were encouraged to work in multidisciplinary teams including epidemiologists, statisticians, and toxicologists. They were asked to address the following qualitative and quantitative questions in their analyses:

Which exposures potentially contributed to the outcome? Are there any that did not? (qualitative).
How much did the exposures potentially contribute to the outcome? (quantitative).
Was there evidence of “interaction?” Be explicit with your definition of interaction [toxicologists, epidemiologists, and biostatisticians tend to think about this quite differently (Howard and Webster 2013)].
What was the effect of joint and cumulative exposure to the mixture? (qualitative).
What is the estimate of the function *Y* = *f*(*X*_1_,…,*X*_p_)? (quantitative).

Workshop participants were also asked to provide specific details about their methodologies and how their assumptions may have influenced the results. These included providing a basic overview of the method(s) used, the rationale for using their approach(es), any transformation or preparation of the data necessary to using the approach(es), and assumptions inherent to the approach(es) and built into the model (e.g., departures from linearity, dose–response shapes, interactions, modifiers, and different potencies for exposures). Participants were also asked to include information about the statistical software they used and to provide the statistical code in their analysis (e.g., R, SAS). They were encouraged to state whether or not they used an existing package or procedure and identify if they had to significantly modify an existing package or procedure, or develop completely new code. The statistical code submitted by participants is available on the workshop’s web site (http://www.niehs.nih.gov/about/visiting/events/pastmtg/2015/statistical/).

Finally, participants were asked to compare the outcomes of their analyses to the correct answers (i.e., truth) associated with the two simulated data sets. If they did not achieve the correct answers for either data set, they were asked to speculate as to why this might have occurred and if changing assumptions would have enabled them to reach the correct result. In addition, the participants were requested to summarize the main strengths and weaknesses of their approach, note any particular challenges they encountered during their analysis (e.g., lack of toxicity data information, limitations in number of exposures that could be evaluated at one time), and recommend next steps.

## Discussion

Numerous statistical approaches were proposed at this workshop and can be categorized as classification and prediction, exposure–response surface estimation, variable selection, and variable shrinkage strategies (Table 1). In general, most of these techniques involved reduction or summation of the exposures in some way. For comparison purposes, some investigators evaluated the commonly implemented linear regression (ordinary least squares) approach. All methods were applied to both the simulated datasets 1 and 2 and some were applied to the real-world dataset.

**Table 1:** Examples of approaches presented at the NIEHS workshop “Statistical Approaches for Assessing Health Effects of Environmental Chemical Mixtures in Epidemiology Studies.”

Method	Category
Single chemical analysis	Classic linear regression (ordinary least squares)
Multiple regression	Classic linear regression (ordinary least squares)
Visualization, structural equation modeling (SEM), and principal component analysis (PCA)	Classification and prediction
Informed sparse PCA and segmented regression	Classification and prediction
Bayesian g-formula	Classification and prediction
PCA	Classification and prediction
Classification and regression trees (CART)	Classification and prediction
Bayesian profile regression	Classification and prediction
Random forest	Classification and prediction
Multivariate adaptive regression splines (MARS)	Classification and prediction
Bayesian non-parametric regression	Classification and prediction
Bayesian additive regression trees (BART) and negative sparse PCA (NSPCA)	Classification and prediction
Conformal predictions	Classification and prediction
Bayesian kernel machine regression (BKMR)	Exposure–response surface estimation
Building Bayesian networks	Exposure–response surface estimation
Exposure surface smoothing (ESS)	Exposure–response surface estimation
Modes of action (results presented for *Z* = 0 strata)	Other
Feasible solution algorithm (FSA)	Other
Exploratory data analysis (EDA)	Other
Novel approach and least-angle regression (LARS)	Variable selection
Machine learning	Variable selection
Two-step variable selection and least absolute shrinkage and selection operator (LASSO)	Variable selection
Two-step shrinkage-based regression	Variable selection
Factor mixture models	Variable selection
Subset and bootstrap	Variable selection
Variable selection regression (VSR)	Variable selection
Bayesian estimation of weighted sum	Variable shrinkage strategies
Shrinkage methods (LASSO/LARS)	Variable shrinkage strategies
Weighted quantile sum regression (WQS)	Variable shrinkage strategies
LASSO	Variable shrinkage strategies

Several general observations emerged from the discussion of these approaches. First, participants agreed that no one statistical approach seemed to perform better than another at the qualitative level for the simulated data sets. Rather, there was extensive variability across the methods and less alignment with the correct answers (i.e., truth) with increasing data complexity (i.e., simulated data set 1 was less complex than simulated data set 2). The various approaches also differed in their ability to address collinearity or correlated variables, interaction between exposures, and model assumptions. Second, many methods included some form of variable and data reduction or transformation, either prior to or while conducting the analysis. Third, there is a need to define specific types of scientific questions and hypotheses related to chemical mixtures that can be addressed by epidemiologic studies (Braun et al. 2016a). More specifically, a statistical method should be chosen based upon a specific scientific question, and the use of complementary methods should be considered when exploring scientific hypotheses. The fact that no one statistical approach appeared to perform better than another may be related the fact that the organizers did not initially pose specific study questions for the analysis. In addition, the way in which the outcomes of interest were conceptualized and analyzed varied among the participants. The organizers designed the data sets with the assumption of prediction, such that the correct and incorrect answers could be easily determined, and it appears that most of the workshop participants also assumed a predictive model approach because no specific hypotheses were identified. Fourth, the limitations of the data analyzed in this workshop must be recognized and addressed to the extent possible. The simulated data sets contained continuous exposure variables, but real-world data often include categorical data. The data sets also contained a restricted number of observations (small sample size) limiting statistical power for some methods as well as other issues inherent in many epidemiologic studies (e.g., co-pollutant correlation, paucity of relevant toxicology data, and insufficient information on potential confounding variables). These issues may be addressed with larger detailed data sets (e.g., larger and more complex simulated data sets and consortium-based or pooled data); existing statistical strategies (e.g., imputation); more collaboration between epidemiologists, toxicologists, and statisticians; or the development of novel methods.

## Conclusions and Future Directions

This workshop and its format were unique and novel in the field of environmental chemical mixtures and epidemiology, because participants were asked to conduct statistical analyses of specific model data sets and to compare their results to other statistical approaches. Based on the attendance, number of abstracts submitted, and enthusiastic discussion at the workshop, this format was successful in bringing statisticians and epidemiologists together to work on a common problem. The questions participants were asked to address in their analyses helped focus the discussion on the desired outcome—specifically, “Which exposures contributed to the outcome?” and “Are there any that did not?” Based on the results from the presentations and abstracts, a significant amount of variability between the methods was evident. Therefore, a useful future activity would be to systematically characterize the variation in results across methods that are sufficiently comparable to effect estimation and statistical significance. Based on the workshop discussions and comparisons across currently used methods, further development of methods is needed to adequately determine the health effects of mixtures and combined exposures. We encourage the ongoing use of the posted simulated data sets, to facilitate collaboration across environmental health disciplines and improve our understanding of the health impacts of chemical mixtures.
